# Speech Rhythm Variation in Early-Stage Parkinson's Disease: A Study on Different Speaking Tasks

**DOI:** 10.3389/fpsyg.2021.668291

**Published:** 2021-06-14

**Authors:** Marta Maffia, Rosa De Micco, Massimo Pettorino, Mattia Siciliano, Alessandro Tessitore, Anna De Meo

**Affiliations:** ^1^Department of Literary, Linguistics and Comparative Studies, University “L'Orientale, ” Naples, Italy; ^2^Department of Advanced Medical and Surgical Sciences, University of Campania “Luigi Vanvitelli, ” Naples, Italy; ^3^Department of Psychology, University of Campania “Luigi Vanvitelli, ” Caserta, Italy

**Keywords:** Parkinson's disease, early-stage, speech rhythm, acoustic analysis, speaking tasks

## Abstract

Patients with Parkinson's disease (PD) usually reveal speech disorders and, among other symptoms, the alteration of speech rhythm. The purpose of this study is twofold: (1) to test the validity of two acoustic parameters—%V, vowel percentage and VtoV, the mean interval between two consecutive vowel onset points—for the identification of rhythm variation in early-stage PD speech and (2) to analyze the effect of PD on speech rhythm in two different speaking tasks: reading passage and monolog. A group of 20 patients with early-stage PD was involved in this study and compared with 20 age- and sex-matched healthy controls (HCs). The results of the acoustic analysis confirmed that %V is a useful cue for early-stage PD speech characterization, having significantly higher values in the production of patients with PD than the values in HC speech. A simple speaking task, such as the reading task, was found to be more effective than spontaneous speech in the detection of rhythmic variations.

## Introduction

Parkinson's disease (PD) is recognized as the second most common neurodegenerative disorder after Alzheimer's disease, with a point prevalence ranging from 0.25 to 4% between the age of 65 and 80 (de Lau and Breteler, 2006). Degeneration of nigrostriatal dopaminergic neurons, which results in the disruption of basal ganglia-thalamo-cortical loops, underlies the classical motor signs and symptoms of PD (i.e., bradykinesia, rigidity, tremor, and postural instability).

The physiological and anatomical changes caused by dopaminergic deficits also affect the three major anatomic subsystems, namely, the respiratory, phonatory, and articulatory systems, governing speech motor control. Typically, 70–90% of patients reveal a disordered oral communication (Darley et al., 1969a; Logemann et al., 1978; Stewart et al., 1995), with the most common speech abnormalities involving hypophonia (or reduced loudness), changes in voice quality (breathy and/or harsh voice), narrow pitch variability, imprecise articulation, and hesitant and disfluent speech (Darley et al., 1969b; Ramig et al., 2008). Interestingly, early speech difficulties in PD have been associated with a less benign clinical phenotype as well as with a higher risk to develop cognitive impairment over time (Polychronis et al., 2019).

At the segmental level, impairment in articulating vowels and consonants, as a consequence of the hypokinesia and the resulting decreased amplitude of motility of the lips, tongue, and jaw, has been widely documented in PD (Forrest et al., 1989; Robertson and Hammerstadt, 1996). It has been shown that a reduction in the vowel space area (VSA) of patients with PD can be predictive of the progression of disease (Skodda et al., 2012), and the degree of imprecision of vowel articulation has been observed in the speaking task of sentence repetition (Sapir et al., 2007, 2010), reading passage (Skodda et al., 2011), and sustained prolongation of single vowels (Eliasova et al., 2013). Rusz et al. (2013) analyzed the vowel articulation across various speaking tasks in a group of 20 Czech patients with early-stage PD prior to pharmacotherapy and found a lowered VSA and abnormalities in formant centralizations measured by the Vowel Articulation Index (VAI) across all speaking tasks with the exception of sustained phonation and with the greatest alteration during spontaneous speech.

At the suprasegmental level of speech, an abnormal pitch variability was extensively observed in patients with PD (Darley et al., 1969b; Stewart et al., 1995; Kent and Kim, 2003; Pinto et al., 2004; Goberman et al., 2005; MacPherson et al., 2011; Skodda et al., 2011).

Furthermore, one of the most common symptoms is the alteration of speech rhythm and rate and speech/pause ratio, as part of a more “general dysrhythmia,” which has been often reported in patients with PD tested also in nonverbal rhythmic tasks, such as finger tapping and gait (Dalla Bella et al., 2018; De Cock et al., 2018; Puyjarinet et al., 2018, 2019).

While there is an agreement on the impact of PD on prosody, the available data in the literature do not highlight a uniform pattern of rhythm and speech/articulation rate alteration in patients with PD. Some authors (Logemann et al., 1978; Ludlow et al., 1987) observed a significantly reduced speech rate in the so-called diadochokinetic (DDK) tasks, in which patients were asked to repeat a series of syllables (/pa/, /ta/, /ka/) at a rapid pace, while others reported the opposite effect (Hirose et al., 1982; Ackermann et al., 1995, 1997). The abnormal speech rate has also been observed with diverging results during tasks used to elicit and evaluate continuous speech (usually reading tasks, as in the studies by Canter, 1963; Gräber et al., 2002; Martínez-Sánchez et al., 2016). Other studies, however, have found no intergroup differences between patients with PD and healthy individuals both in speech rate (Duez, 2006a,b) and in articulation rate (Skodda and Schlegel, 2008).

Some acoustic studies focused on abnormalities of duration of speech segments and indicated a complex and different effect of PD on consonants and vowels. Consonants were shown to be shortened in PD speech compared with healthy control (HC) speech (Canter, 1963; Duez, 2006a,b; Maffia et al., 2020), whereas vowels were found to be both longer (McRae et al., 2002; Duez, 2006a,b; Maffia et al., 2020) and shorter (Forrest et al., 1989; Baudelle et al., 2003) in speech of patients or of the same duration as in the control production (Bunton and Weismer, 2001).

Different rhythmic metrics were used to describe Parkinsonian speech. In a study on American English patients with PD, Liss et al. (2009) used the standard deviation (SD) of vocalic intervals over a sentence (ΔV), the SD of consonantal intervals over a sentence (ΔC), the percentage of vocalic intervals (%V), the rate-normalized SD of vocalic and consonantal intervals (VarcoV and VarcoC), the pairwise variability indices (nPVI-v and rPVI-c), and the articulation rate with the purpose of identifying with a high level of accuracy the rhythmic variations in PD productions compared with healthy speech samples. In this study, the parameter %V was found to be one of the most effective parameters in characterizing dysarthric speech.

Research on Italian patients with mild-to-severe PD confirmed the usefulness of %V in association with VtoV, the mean interval between two consecutive vowel onset points (VOPs), for the detection of rhythmic variation in PD read speech, compared with healthy productions (Pettorino et al., 2016, 2017).

To diagram an utterance on the basis of %V and VtoV has been demonstrated to be a very effective tool to represent its rhythmic characteristics (Pettorino et al., 2013). VOPs, indeed, represent the signal discontinuities that guide listeners in the perception of rhythm (Barbosa, 2002; Barbosa et al., 2005); the smaller the VtoV is, the closer the vowels are to each other and the more accelerated the speech is perceived. From this perspective, VtoV could be considered as the perceptual counterpart of the articulation rate; if the latter refers to speech production and it is calculated in terms of syllables, VtoV is a measure of the perceived rhythm (Pettorino et al., 2013).

On the contrary, the %V parameter is independent of the articulation rate: “the greater the vowel percentage, the greater the continuity of the speech signal perceived by the listener. Conversely, a greater consonant interruption will determine the perception of a less continuous speech: this is what, in musical terms, goes under the name of *legato* and *staccato*” (Pettorino et al., 2017, p. 3,173).

The %V/VtoV metrics were also applied in a case study, in which the speech rhythm of the Canadian actor Michael J. Fox, diagnosed with young-onset PD in 1991, was diachronically analyzed (Pettorino et al., 2018). The data showed an abnormal increase in %V in the actor's speech as early as 1986, which was 5 years before the first motor symptoms of the disease appeared.

As in the case of Michael J. Fox, speech disorders in the early stages of PD are often mild and barely perceptible to others or even to the speakers themselves, with speech intelligibility being limitedly compromised at the disease onset. Nevertheless, numerous studies have tried to find acoustic parameters that can serve as markers of PD or indices for its progression (among the others, Rusz et al., 2011 and Rusz et al., 2013).

The results of experimental studies on different languages suggested, indeed, that the instrumental observation of variation of some acoustic parameters in the speech of patients with PD may potentially provide a sustainable and noninvasive diagnostic tool, in support of clinical assessment, even at the very early stages of the disease, when the neurodegeneration is yet started and spread throughout the nervous system but still there are no other motor signs (King et al., 1994; Holmes et al., 2000; Cohen, 2003; Harel et al., 2004).

Even if the research results in this direction are promising, some limitations, such as a lack of a widely accepted and shared methodology for speech data collection and small subject pools (Dimauro et al., 2017), exist. A study conducted by (Weismer, 1984) suggested, for example, that the degree of articulatory alteration may vary between simple and complex tasks produced by patients with PD, due to the fact that simple speaking tasks (e.g., sentence repetition or reading passage) do not require the full attention of speakers and are more automatic than structured and complex tasks, such as spontaneous monologs. Nevertheless, minimal effort has been given to explore the severity of articulatory or prosodic variation in PD speech under various speaking tasks (Rusz et al., 2013; Juste and Andrade, 2017; Lowit et al., 2018). In particular, to the knowledge of authors, there are no studies focused on this specific topic conducted on Italian PD speech.

In continuity with previous research on Italian PD speech rhythm and in order to address the methodological issues presented earlier, this study is designed to answer the following research questions:

- Can speech rhythm changes, evaluated by the calculation of the two acoustic parameters %V and VtoV, be found in the early stages of PD?
- Which speaking task between reading passage and monolog is most sensitive to speech rhythm variation in PD?

## Materials and Methods

### Participants

The data for this study were collected from a total of 40 Italian native speakers residing in the Campania region (south of Italy). A group of 20 patients with early-stage PD was recruited at the Movement Disorders Unit of the First Division of Neurology at the University of Campania “Luigi Vanvitelli” (Naples). The diagnosis of PD was based on the modified diagnostic criteria of the UK Parkinson's Disease Society Brain Bank (Gibb and Lees, 1988). Inclusion criteria were as follows: (1) PD onset after the age of 40 years, to exclude early-onset parkinsonism; (2) a modified Hoehn and Yahr (mH&Y) stage ≤ 2.5; and (3) disease duration ≤ 4 years. Exclusion criteria were as follows: (1) relevant cognitive impairment associated with PD according to consensus criteria; (2) major depression, minor depression, and dysthymic disorder according to DSM-IV criteria; and (3) any other neurological disorder or clinically significant medical condition. A group of 20 age- and sex-matched HCs with no history of neurological or speech disorders was also enrolled in this study. All subjects gave written consent to the data collection procedure.

#### Clinical Assessment

All patients underwent an extensive motor and non-motor assessment with validated PD-related scales. The disease severity was assessed by means of the mH&Y and the Unified Parkinson's Disease Rating Scale part III (UPDRS III).

UPDRS III was also used to assess the presence of clinically significant speech difficulties according to Item 3.1 (Speech) ≥ 1. This item is a clinician-based scale consisting of 5 scores, rating between 0 (normal) and 4 (most severe impairment).

Global cognitive functioning was assessed with the Montreal Cognitive Assessment (MoCA, Folstein et al., 1975). Moreover, depressive symptoms were rated by means of the Beck Depression Inventory (Beck et al., 1961). Finally, daily total amount of dopaminergic medication (i.e., Levodopa Equivalent Daily Dose) was computed using an algorithm adapted from the study by Tomlinson et al. (2010).

The clinical and demographical data of patients with PD, together with the characteristics of the HC group, are summarized in Table 1.

**Table 1:** Demographic and clinical features of patients with PD and HC.

		**HC (*n* = 20)** ** Mean ± SD**	**PD (*n* = 20)** ** Mean ± SD**	***p*-value**
Demographic data	Age	64.8 ± 5.9	63.8 ± 10.9	*0.713*
	Sex (M/F)	8/12	12/8	*0.205*
	Disease duration (months)	-	31.9 ± 17.1	-
Clinical data	mH&Y stage	-	2.1 ± 0.4	-
	UPDRS III	-	24.0 ± 7.6	-
	Item 3.1 (Speech)	-	0.7 ± 0.5	
	MoCA total	-	22.4 ± 4.0	-
	BDI	-	5.2 ± 3.6	-
	LEDD total	-	202.5 ± 157.8	-
	LEDD DA	-	30.0 ± 76.6	-

*PD, Parkinson's disease; HC, healthy control; mH&Y, modified Hoehn&Yahr; UPDRS, Unified Parkinson's Disease Rating Scale; MoCA, Montreal Cognitive Assessment; BDI, Beck Depression Inventory; LEDD, Levodopa Equivalent Daily Dose; DA, dopamine-agonist*.

### Speech Data Collection

Each subject was instructed to read aloud an expository text in Italian comprising about 350 syllables and 175 words from a printed sheet. The text was accurately chosen for presenting a high level of readability (Gunning Fog index: 6 and Gulpease index: 70), simple morphosyntactic structures, a large number of high-frequency words (94% on the total), and a very common topic, i.e., comparing eating habits of the past with those of the present. Unlike other studies on Italian dysarthric speech, in which non-sense or improbable texts were used in reading tasks (see, e.g., Dimauro et al., 2017), the choice to propose a realistic text was made in order to avoid reading difficulties due to the noncomprehension of the written passage, especially in the case of low-educated subjects.

Moreover, the participants were asked to produce an extemporaneous monolog of minimum 1 min, talking about positive and negative aspects of the place where they lived at the moment of data collection.

All the subjects were encouraged to speak in their normal, conversational voice, as spontaneously as possible, and at comfortable loudness. The speech samples were recorded on a standard personal computer in a quiet room of University “Luigi Vanvitelli,” by means of the software Praat (Boersma and Weenink, 2021) at a 44,100 Hz sampling rate. A total duration of about 105 min of PD and healthy speech was recorded.

Information about linguistic repertoires and uses for each speaker was also obtained with the administration of a sociolinguistic questionnaire.

Patients performed the speech assessment while taking their regular dopaminergic medications.

### Speech Analysis

**Figure 1:**
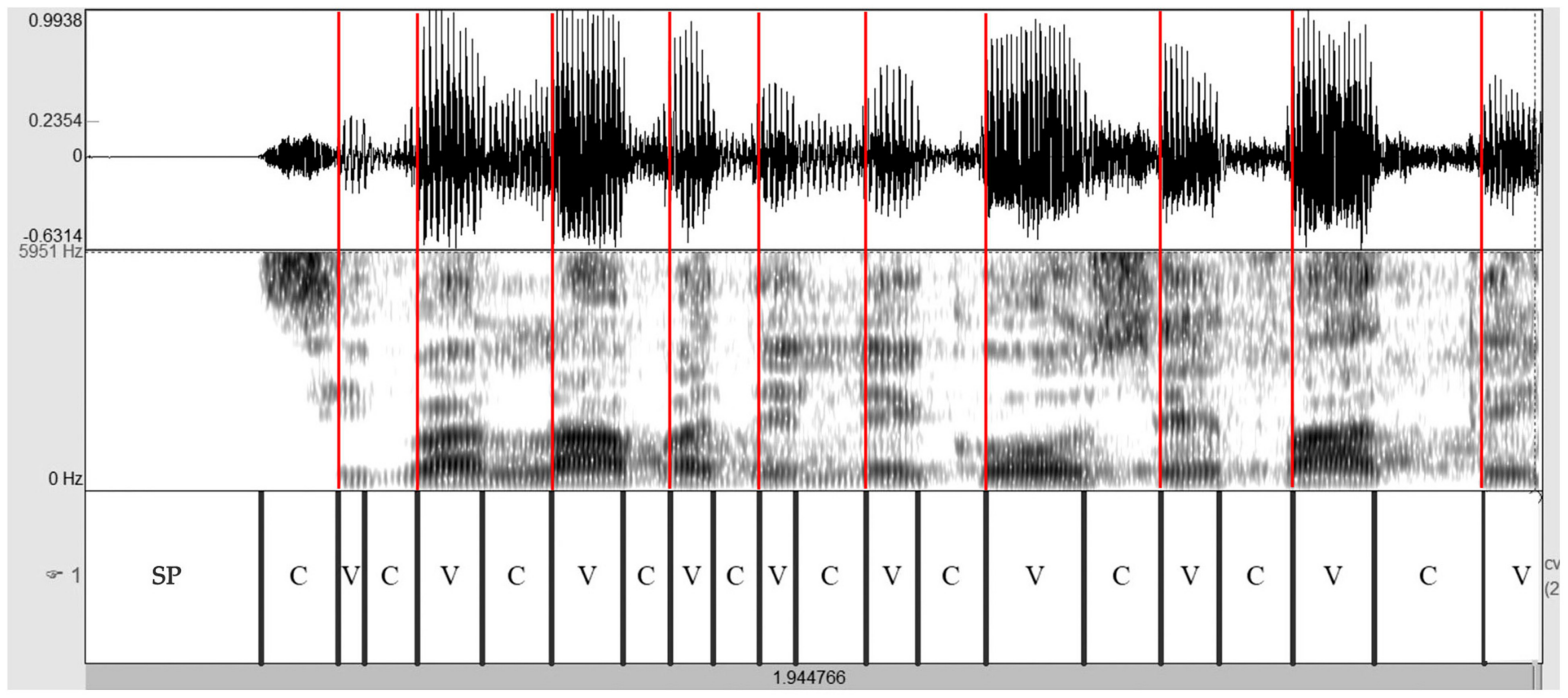
Spectrogram of the utterance “*si parlava delle cose fatte*” (PD male voice—read speech). Red lines are placed in correspondence with the VOPs. Based on the spectrographic tracing and even more from the amplitude trend of the complex wave, it is clear that these instants coincide with the discontinuity points of the signal, on which the perception of rhythm is based. PD, Parkinson's disease; C, consonantal interval; V, vocalic interval; SP, silent pause; VOPs, vowel onset points.

**Figure 2:**
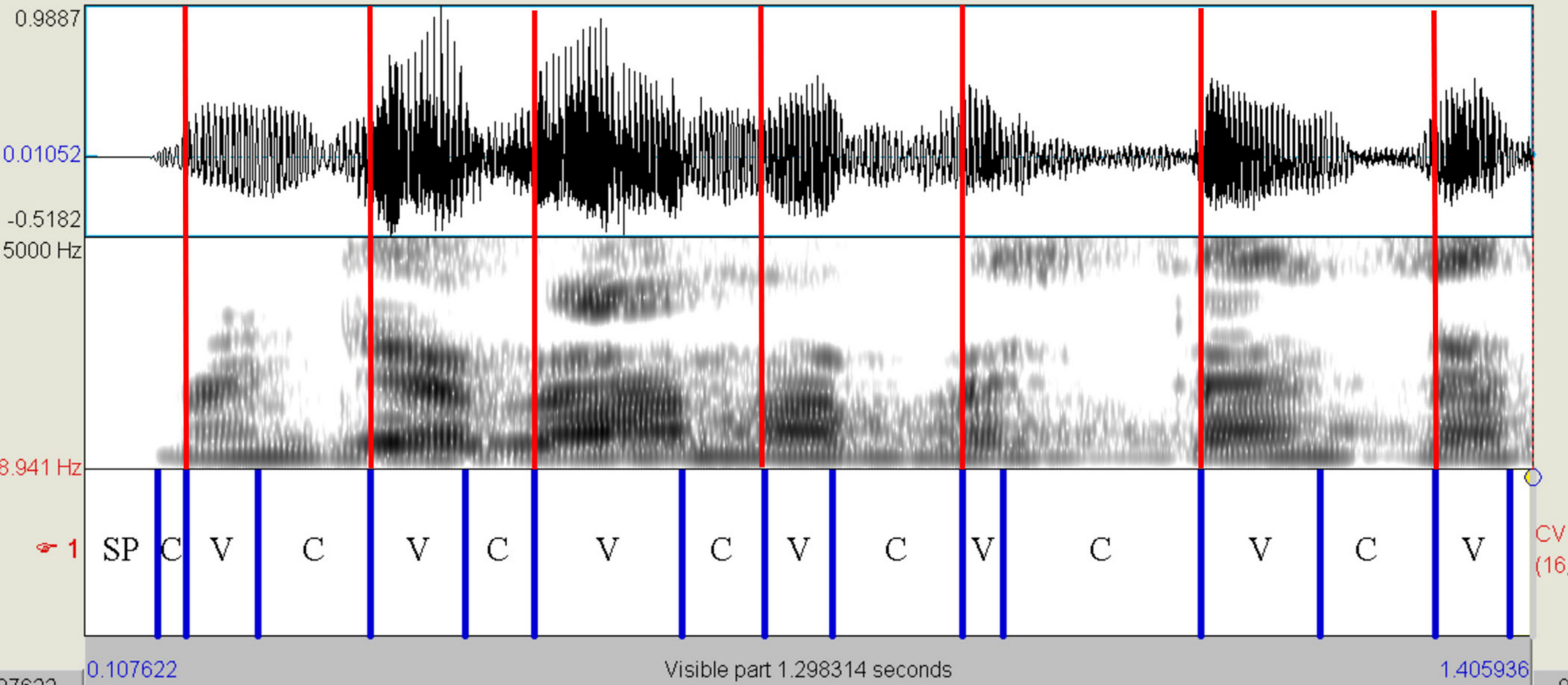
Spectrogram of the utterance “*mangiavamo abbastanza*” (PD female voice—read speech). Red lines are placed in correspondence with the VOPs. The first and penultimate vowels [a] are followed by the nasal consonant [n]. In both cases, the V interval includes the nasalized part of the vowel. PD, Parkinson's disease; C, consonantal interval; V, vocalic interval; SP, silent pause; VOPs, vowel onset points.

**Figure 3:**
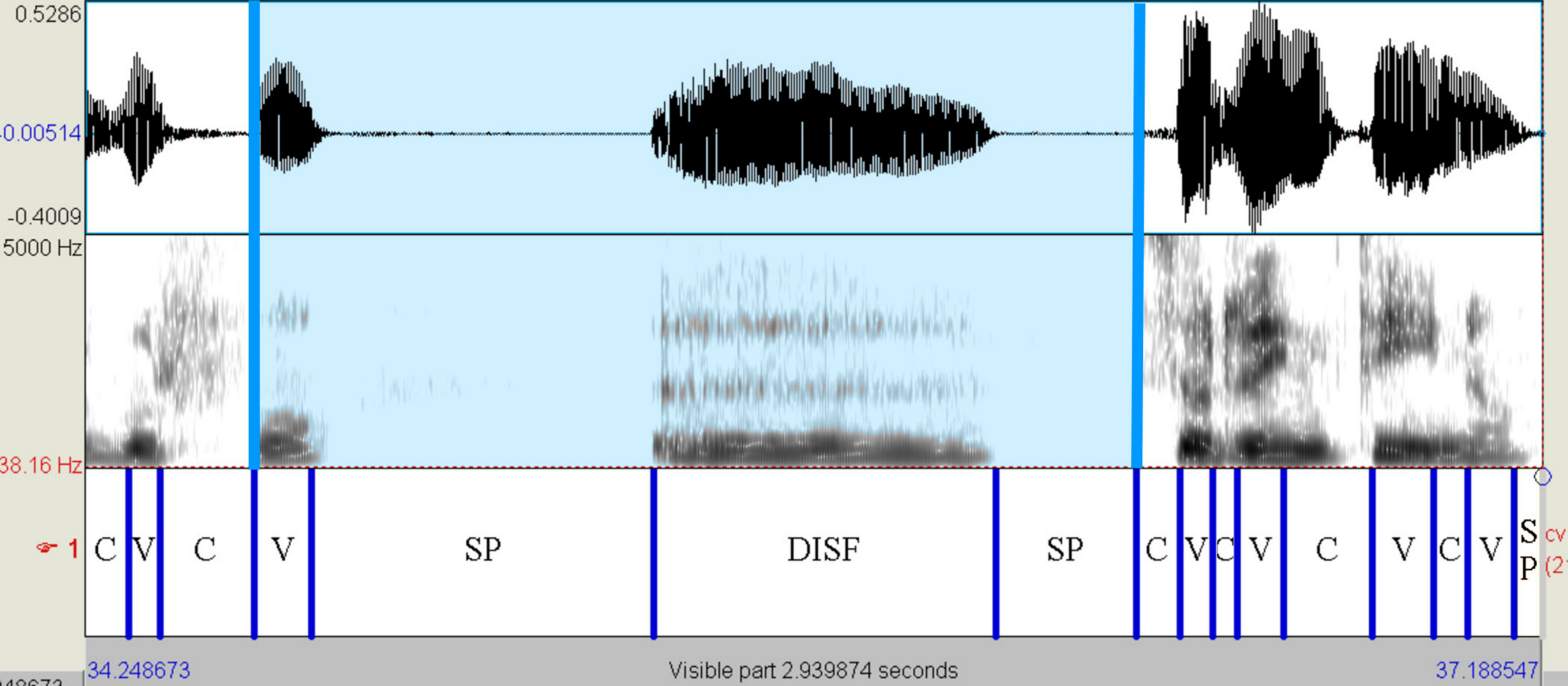
Spectrogram of the utterance “*golfo* <sp> [e:] <sp> *sorrentino*” (HC female voice—monolog). The presence of disfluencies, a vocalization in this case, testifies to the spontaneous nature of the utterance. The blue-colored interval, including prepausal vowel, disfluency, and silent pauses, has not been considered in determining the VtoV value. On the contrary, the prepausal vowel was taken into account in calculating the %V. HC, healthy control; C, consonantal interval; V, vocalic interval; SP, silent pause; DISF, disfluency.

The read and spontaneous speech samples were manually labeled on Praat to identify consonantal (C) and vocalic (V) intervals through the visual inspection of speech spectrograms and waveforms (see Figures 1–3 for some samples). Approximants were completely avoided in the text used in the reading task and treated as vowels when occurring in monologs. Diphthongs were always considered as a single vocalic interval. In V + nasal consonant sequences, the nasalized portion of the vowel was assigned to the V interval. As for initial voiced stop consonants, the first boundary was considered to be the onset of the glottal pulses. Post-pausal voiceless plosives were assigned a duration equal to the mean value of single plosives in the same utterance.

Once extracted the durations of all consonantal and vocalic portions, the values of %V and VtoV for each read and spontaneous speech sample were obtained using a Praat script. Disfluencies and silent pauses were not considered in the calculation of these two rhythmic parameters.

However, silences, filled pauses, hesitations, false starts, repairs, prolongations, and any kind of disfluencies occurring in both read and spontaneous speech were also labeled, and speech time composition (i.e., percentage of silence, disfluency, and fluent speech) for each subject and in both speaking tasks was calculated. All the segments whose duration exceeded 50% of mean internal duration for that specific class of sound in the specific speech sample were considered as lengthening: in these cases, a portion equal to the mean internal duration of that sound in that speech sample was labeled as V or C, and the remaining portion was labeled as disfluency.

The labeling of speech samples was independently conducted by two of the authors, and the points of disagreement were discussed and resolved by consensus.

### Statistical Analysis of Clinical and Speech Data

Independent samples *t*-test or chi-squared test (categorical variables) was used to compare demographic data in PD and HC groups, as appropriate. A two-way repeated measures ANOVA was performed on the two dependent variables of speech analysis (%V and VtoV), considering the kind of speaking task (i.e., reading and monolog) as within-subject factor and the group (HC and PD) as a between-subjects factor (i.e., independent variables). *Post-hoc* pairwise comparisons were used to compare PD and HC data in each speaking task (independent samples) and speech features in the two speaking conditions within each group (paired samples). The level of significance was set as *p* < 0.05. Cohen's *d* value was calculated to assess the effect size of pairwise comparisons using pooled variance.

Moreover, bivariate correlations were performed to test the association between the subscores of the UPDRS III and speech data. Pearson's correlation coefficients were computed considering a *p*-value <0.05 as statistically significant. The analyses were performed with SPSS version 23 (SPSS Inc., Chicago, IL, USA).

## Results

### Clinical Data

Twenty patients (12 males and 8 females) with early-stage PD (mean ± SD mH&Y stage: 2.1 ± 0.4; mean ± SD UPDRS III 24.0 ± 7.6) were enrolled in this study. The age of patients was in the range from 41 to 81 years (mean: 63.8 years; SD: 10.9 years). Thirteen patients with PD exhibited mild-to-moderate speech difficulty (Item 3.1 Speech = 1 or 2) and seven patients with PD had no speech difficulty (Item 3.1 Speech = 0). There were no patients with PD who presented with moderate–severe speech difficulty (Item 3.1 Speech ≥ 3).

### Speech Rhythm

**Figure 4:**
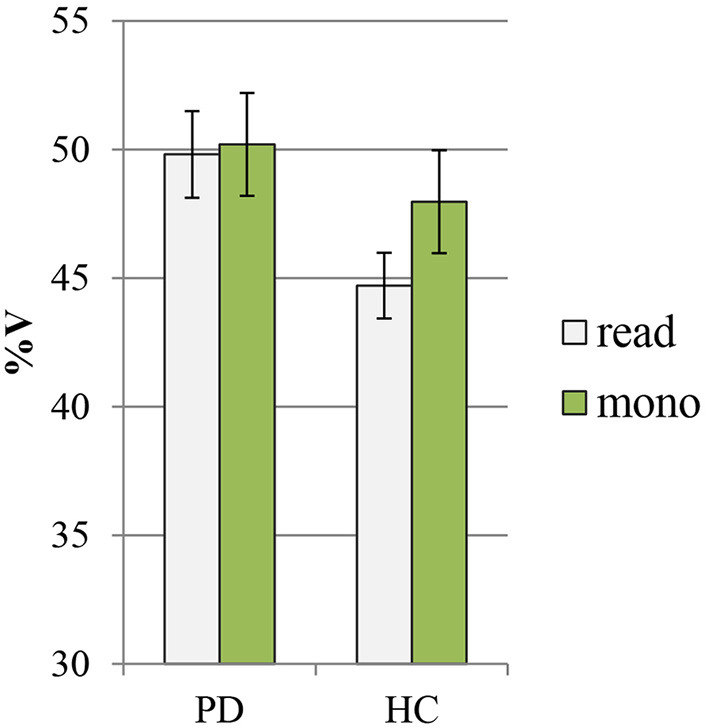
Mean values of %V in PD and HC productions (read: reading task; mono: monolog). PD, Parkinson's disease; HC, healthy control.

**Figure 5:**
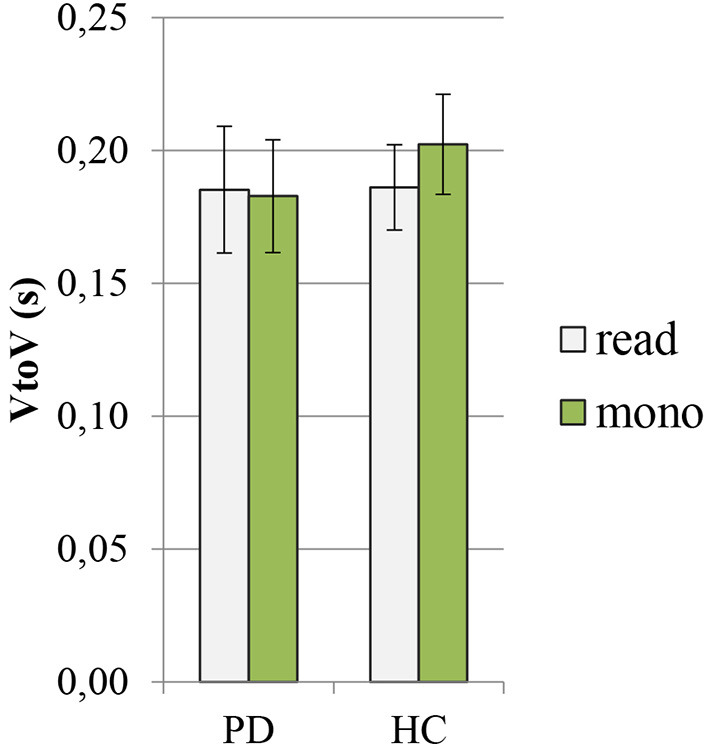
Mean values of VtoV (s) in PD and HC productions (read: reading task; mono: monolog). PD, Parkinson's disease; HC, healthy control.

In Figures 4, 5, the mean values of the two rhythmic parameters, %V and VtoV (s), in PD and HC read and spontaneous speech are illustrated.

The ANOVA showed a significant effect of both group and speaking task on %V values (group: *F* = 72.3, *p* < 0.00000000001, η^2^ = 0.39; speaking task: *F* = 20.7, *p* = 0.00002, η^2^ = 0.11). According to pairwise comparisons, the PD group displayed a distinctly higher %V in comparison with HC group both in the reading task (PD: 49.8 ± 1.7 vs. HC: 44.7 ± 1.3, *p* < 0.0000001, Cohen's *d* = 3) and in the monolog (PD: 50.2 ± 2 vs. HC: 47.9 ± 2.4, *p* = 0.002, Cohen's *d* = 0.9). From an intragroup perspective, the PD data in the two tasks show very similar mean values of %V, while the variable task produces significant changes only in the mean %V of HC productions, with higher values displayed in the spontaneous than in the read speech (read: 44.7 ± 1.3 vs. mono: 47.9 ± 2.4, *p* = 0.000003, Cohen's *d* = 1.8).

In the case of VtoV values, the effect of group was found to be significant (*F* = 5.02, *p* = 0.02, η^2^ = 0.05), while that of the speaking task was not significant (*F* = 1.81, *p* = 0.18, η^2^ = 0.02). The pairwise comparisons showed significant intergroup differences only in the case of spontaneous speech, with higher values found in HC than in PD (PD: 0.183 ± 0.02 vs. HC: 0.202 ± 0.02, *p* = 0.003, Cohen's *d* = 0.9). The variable speaking task has an effect on the duration of VtoV mean interval only in the HC group. It was found to be significantly longer in HC monologs than in the reading task (read: 0.186 ± 0.02 vs. mono: 0.202 ± 0.02, *p* = 0.005, Cohen's *d* = 0.8).

A preliminary analysis was conducted to compare performances in the two speaking tasks between patients with PD with and without clinically significant speech difficulties. We found statistically significant higher VtoV values only in the case of monolog in patients with PD with speech difficulties (*p* = 0.04).

### Speaking Tasks and Speech Time Composition

**Figure 6:**
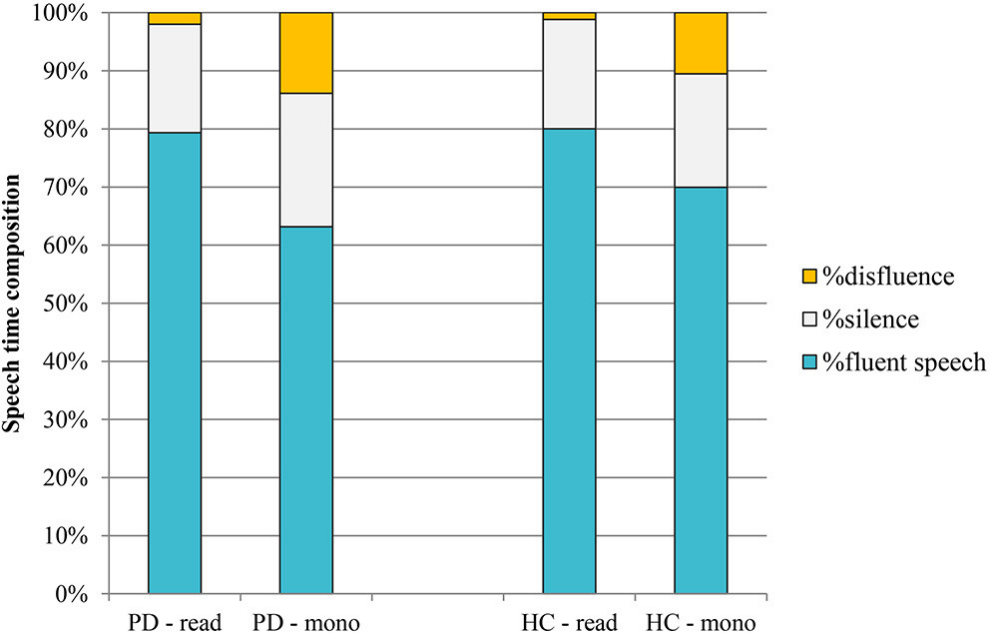
Mean speech time composition in PD and HC productions (read: reading task; mono: monolog). PD, Parkinson's disease; HC, healthy control.

Figure 6 shows the mean composition of the utterance for the two different speaking tasks in PD and HC speakers.

As the graph in the figure shows, no significant differences were observed between the two groups of speakers in terms of speech time composition. In the reading task, percentages are very similar in PD and HC; in the case of monologs, PD productions are characterized by a slightly higher percentage of silence (23 vs. 20% in HC) and of disfluency (14 vs. 11% in HC).

As expected, the most evident result is related to the effect of the speaking task on the composition of the utterance: the mean increase of the percentage of disfluency in the monologs of both groups of speakers in comparison with the reading tasks (from 2 to 14% in PD; from 1 to 11% in HC) and the subsequent reduction of the fluent speech percentage.

### Correlation Analysis

A positive correlation was found between the scores given by clinicians to Item 3.1 of the UPDRS, assessing the presence and the degree of speech impairment on a perceptual basis, and VtoV mean values in the case of the monologs of patients with PD (*R* = 0.481; *p* = 0.031). It means that the more the speech of the patient was perceived as decelerated, the more impaired it was during the clinical assessment. No other correlations were found between UPDRS III scores and speech analysis data.

## Discussion

This study had a twofold objective. First, it aimed at verifying the validity of the two rhythmic parameters, %V and VtoV, for the speech characterization of Italian subjects with early-stage PD. Second, it wanted to determine if a different speaking task (i.e., reading passage or monolog) could produce significant changes in the speech rhythm of patients with PD.

To reach the goals, read and spontaneous speech samples of 20 non-demented patients with early-stage PD were spectro-acoustically analyzed and compared with the same productions of a HC group.

As for the first objective, this study confirms that one of the two parameters, %V, changes significantly in the two groups of speakers, being distinctly higher in PD speech than in HC productions, according to the results of previous studies (Liss et al., 2009; Pettorino et al., 2016, 2017, 2018; Lowit et al., 2018).

It can be supposed that variation in %V values is determined by the motor symptoms characterizing PD, such as the difficulty at initiating movements (akinesia), the slowing of the velocity in the execution of movements once initiated (bradykinesia), and the muscular rigidity. In fact, such motor impairments have different effects on the articulation of vowels and consonants. While vowels are static sounds, which require very limited motor and neuromuscular activity, consonants are dynamic sounds, requiring rapid and synchronized movements of the phonatory organs or, as in the case of fricatives, a continuous control of a specific articulatory configuration and a precise calibration of muscle tension. As a consequence, in the dysarthric speech of patients with PD, vocalic gestures are sustained once they have been started, and the articulatory passage to the consonantal dynamic phase is delayed. This prolonging of the static phase accounts for the greater %V in PD speech with respect to healthy speakers. The alteration of this acoustic parameter even at the initial stages of PD may potentially reflect neuropathophysiological changes that occur very early in the disease course.

Conversely, the articulation rate, expressed by VtoV mean values, does not seem to unambiguously distinguish dysarthric from healthy speech. Significant lower VtoV mean values were found in the spontaneous speech of patients with PD when compared with HC monologs. Nevertheless, according to the correlation analysis results, this parameter seems to be the main perceptive cue for the clinical rating of speech ability of the patient (i.e., UPDRS—Item 3.1).

**Figure 7:**
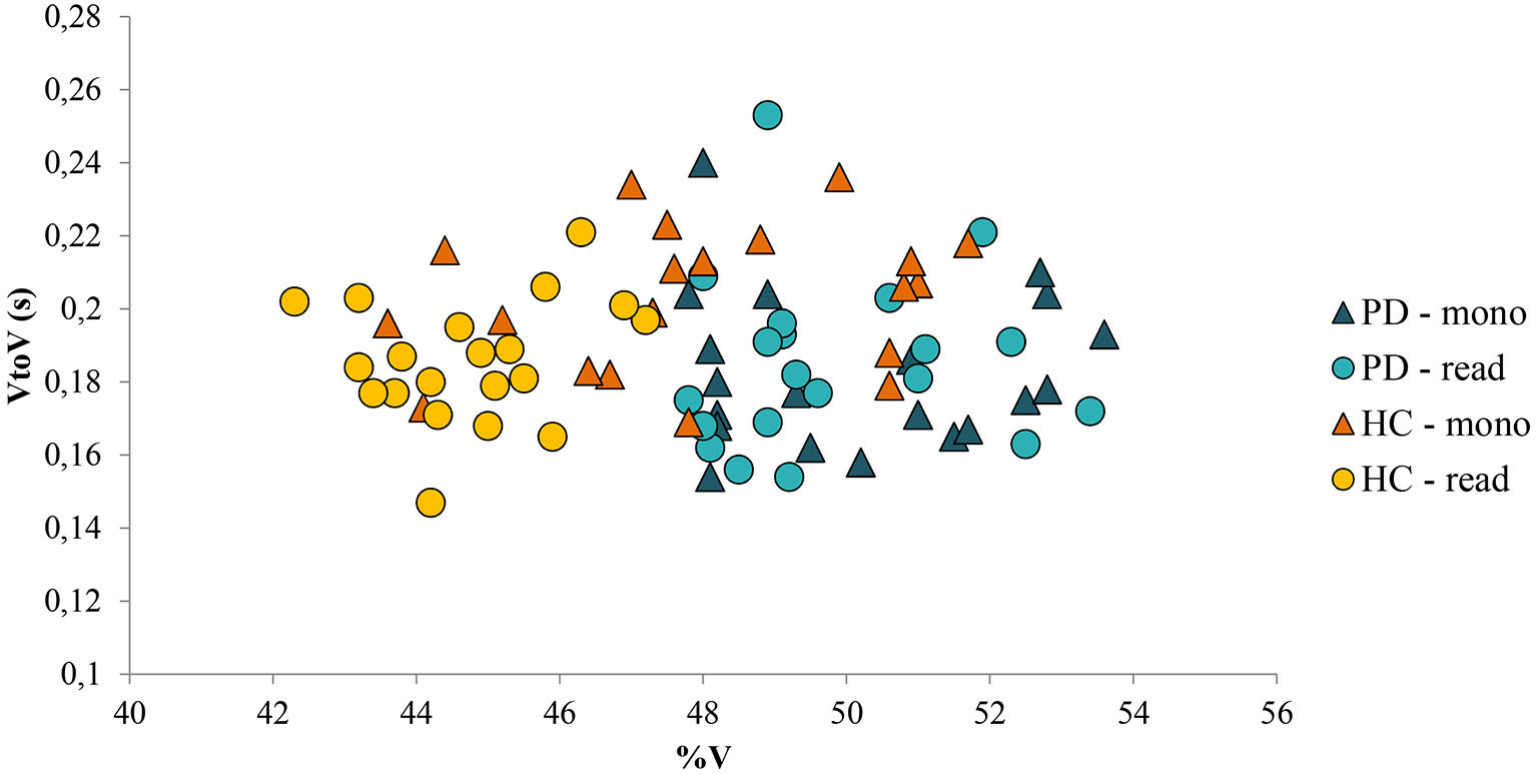
%V values and VtoV mean values (s) for each PD and HC speaker and for both speaking tasks (read: reading task; mono: monolog). PD, Parkinson's disease; HC, healthy control.

Regarding the second objective of this study, no differences were found in the speech rhythm of patients with PD between the reading task and the monolog. In Figure 7, in which %V and VtoV mean values (s) for each speaker and for both speaking tasks are reported, it is possible to notice that the variable kind of task did not produce significant variations in terms of %V and VtoV values in the PD group; the areas covered by the two kinds of dots corresponding to PD productions are completely overlapped. In addition, Figure 7 shows a clear intergroup difference in the distribution of the values along the %V-axis. Although HC and PD areas in the plot partially overlap in the case of monolog, a significant difference between %V values in the two groups and in the two tasks was found. The threshold value for %V can be estimated at ~48%, with the patients with PD having vowel percentages always above it and the control group mostly below it.

In summary, to answer our second research question, both spontaneous and nonspontaneous speech seems to be suitable for the assessment of early changes in speech rhythm associated with PD. In contrast with the results of previous studies (Rusz et al., 2013; Lowit et al., 2018), the reading passage seems to be the task that maximizes the difference between patients with PD and HC productions in terms of %V. On the other hand, this study highlights the fact that it is also possible to use spontaneous speech data to detect rhythmic differences in PD subjects, allowing future larger pooling of data from various sources, with the involvement, for example, of low-educated people.

An unexpected result of this study was the significant variation observed in the speech rhythm of HC participants between the two speaking tasks, with distinctly higher values of both %V and VtoV in the monologs than in the read speech. This result seems to be in contrast to the common definition of spontaneous speech, usually represented by a shorter vowel duration, associated with a faster speech rate. In spontaneous speech, the decrease in the duration is supposed to be the main determinant of vowel reduction, consisting in the consequent occurrence of *target undershoot* (Lindblom, 1963).

Although the different nature of the two speaking tasks was confirmed by data on speech time composition, with all monologs characterized by a higher amount of disfluencies (Figure 6), the results of this study do not confirm the decrease in vowel duration in spontaneous speech both in PD and in HC productions. As shown in Table 2, there are no significant changes in consonantal and vocalic mean durations between the two speaking tasks in the PD group. In the HC group, the higher value previously noticed in VtoV in the case of monolog (Figure 5), corresponding to a reduced articulation rate, has a significant effect only on mean vowel duration. Conversely, the difference between consonantal duration in the two HC speaking tasks is not significant.

**Table 2:** Mean duration of vocalic and consonantal portions in the two speaking tasks performed by PD and HC groups.

		**Reading task Mean ± SD**	**Monolog Mean ± SD**	***p*-value**
HC (*n* = 20)	durV (ms)	81.6 ± 7.9	94.9 ± 11.1	**0.00007**
	durC (ms)	103.2 ± 9.1	107.6 ± 10.2	0.15
PD (*n* = 20)	durV (ms)	91.3 ± 12.1	91.5 ± 11.8	0.96
	durC (ms)	93.5 ± 12.7	93.4 ± 11.4	0.99

*Significant difference is mentioned in bold*.

A possible explanation for these data could be found in the different overall demands and degree of complexity of the two speaking tasks: while in the reading passage the speaker is asked to simply pronounce a ready-made text and he/she has the possibility to provide more attention to articulatory performance, in the spontaneous speech the speaker carries out the complete planning process (Weismer, 1984; Levelt, 1989). In a more cognitively complex speaking task, in which the attention of the speaker is mostly directed to the choice and the planning of what to say, time for language processing is gained on disfluencies, but also, as our data suggest, on vowels, the simplest sounds to be pronounced. This is what seems to happen in the HC group.

Instead, in the dysarthric speech of patients with PD, the speaking task has no effect on %V since the increase of mean vowel duration is already occurring in the read speech, due to the above-mentioned alterations in motor control circuits within the basal ganglia.

## Conclusion

At the moment, the diagnosis of PD is exclusively clinical, with a lack of laboratory and instrumental tests for monitoring the disease evolution and the treatment response. The UPDRS is widely used by neurologists for the evaluation of the disease progression, and specifically, section 3.1 of the scale provides the specialist some tips to rate the speech abilities of patients. According to our preliminary results, it can be supposed that the articulation rate is the main perceptive cue for the clinical identification of speech disorders.

Acoustic measurement of rhythmic parameters, such as VtoV and %V, may give more precise and objective information in support of the clinical assessment. Specifically, it seems that the observation of %V, more than the articulation rate, can be useful as noninvasive procedure to rate the overall clinical speech burden of PD and may also be potentially proposed to monitor the development of dysarthria, which has been associated with a more rapid disease progression.

Furthermore, longitudinal studies on larger PD Italian samples and also on other languages, with different rhythmic patterns, are needed to support and enlarge our observations.

## Data Availability Statement

The raw data supporting the conclusions of this article will be made available by the authors, without undue reservation.

## Ethics Statement

The studies involving human participants were reviewed and approved by Ethical committee of the University of Campania Luigi Vanvitelli, according to the Helsinki Declaration. The patients/participants provided their written informed consent to participate in this study.

## Author Contributions

MM and RD: research project design, collection, classification and interpretation of the data, writing, and final revision of the manuscript. MP, AT, and AD: research project design, interpretation of the data, and final revision of the manuscript. MS: collection and classification of the data and final revision of the manuscript. All authors contributed to the article and approved the submitted version.

## Conflict of Interest

The authors declare that the research was conducted in the absence of any commercial or financial relationships that could be construed as a potential conflict of interest.
